# Identification of microglia-associated hub genes and a CX3CR1/P2RY12 two-gene diagnostic model for cross-subtype epilepsy-associated cortical malformations: integrated transcriptomic analysis with external validation

**DOI:** 10.3389/fnins.2026.1954412

**Published:** 2026-10-08

**Authors:** Hongliang Zeng, Bin Li

**Affiliations:** Ganzhou Hospital-Nanfang Hospital, Southern Medical University (Ganzhou People’s Hospital), Ganzhou, China

**Keywords:** CX3CR1, diagnostic model, focal cortical dysplasia, microglia, neuroinflammation, P2RY12, tuberous sclerosis complex, WGCNA

## Abstract

Focal cortical dysplasia (FCD) and tuberous sclerosis complex (TSC) are major causes of drug-resistant epilepsy, yet shared molecular mechanisms across these structurally distinct malformations remain poorly characterized. We performed an integrated transcriptomic analysis of RNA-seq data from GSE256068 [85 samples: 64 cortical (FCD2a, FCD2b, TSC, controls) and 21 hippocampal (TLE-HS, controls)]. Differential expression analysis with limma identified 865, 1,915, 2,772, and 3,749 DEGs in FCD2a, FCD2b, TSC, and TLE-HS versus controls, respectively, with 49 shared DEGs across all four subtypes. Weighted gene co-expression network analysis (WGCNA) identified the brown and red modules as key disease-associated modules. By intersecting WGCNA hub genes (|GS| > 0.5, |MM| > 0.8) with shared DEGs, we identified 15 hub genes, all functionally associated with microglia/myeloid lineage, including IRF8, TLR7, CX3CR1, P2RY12, C3AR1, and CCR1. Gene set enrichment analysis (GSEA) revealed 15 KEGG pathways consistently enriched across all subtypes, converging on Toll-like receptor, NF-kB, chemokine, and complement signaling. Immune deconvolution using MCP-counter and xCell confirmed elevated monocytic lineage and T cell infiltration in disease cortex, with monocytic scores showing a positive trend with seizure frequency. A two-gene logistic regression model (CX3CR1 + P2RY12), selected by LASSO and random forest, achieved a training AUC of 0.979 and a nested five-fold cross-validation AUC of 0.862 (95% CI: 0.693–1.000), with an external validation AUC of 0.865 (95% CI: 0.681–1.000) in GSE213488. Drug repurposing via Enrichr identified vorinostat (HDAC inhibitor) as a top candidate, and IRF8 was predicted as the master transcriptional regulator. *In vitro*, mTOR activation with MHY1485 preserved P2ry12 expression in BV-2 microglia, whereas LPS stimulation induced its loss, supporting a distinct microglia/myeloid-associated program driven by mTOR hyperactivity. These findings demonstrate that cross-subtype transcriptomic convergence on microglial innate immune activation represents a unifying molecular mechanism in FCD and TSC, and that CX3CR1 and P2RY12 together constitute a two-gene diagnostic classifier with encouraging but preliminary performance with potential clinical utility.

## 1 Introduction

Epilepsy affects approximately 50 million people worldwide, with approximately 30% of patients developing resistance to anti-seizure medications despite optimal pharmacotherapy (Thijs et al., 2019). Focal cortical dysplasia (FCD) and tuberous sclerosis complex (TSC) represent the most common structural etiologies encountered in epilepsy surgery series (Blumcke et al., 2017; Najm et al., 2022). FCD is classified histopathologically into types I, IIa, IIb, and III, characterized by cortical dyslamination, dysmorphic neurons, and balloon cells (Blumcke et al., 2017). TSC, caused by loss-of-function mutations in TSC1 or TSC2, produces cortical tubers that share histological features with FCD type IIb (Curatolo et al., 2015). Despite distinct genetic etiologies, the clinical and histopathological overlap suggests shared downstream molecular mechanisms.

Neuroinflammation – particularly microglial activation – is increasingly recognized as a contributor to epilepsy pathogenesis (Eyo et al., 2017; Vezzani et al., 2019). Activated microglia have been demonstrated in surgical specimens from FCD and TSC patients, and TSPO-PET imaging has revealed microglial activation in epileptogenic foci (Gershen et al., 2015). Pro-inflammatory cytokines including IL-1beta, TNF-alpha, and IL-6 are elevated in cerebrospinal fluid and brain tissue from epilepsy patients (Vezzani et al., 2019). However, the transcriptome-wide molecular programs driving microglial activation across different epilepsy subtypes remain incompletely characterized.

Systems biology approaches, including weighted gene co-expression network analysis (WGCNA) and machine learning-based feature selection, offer powerful frameworks for identifying disease-associated gene modules and diagnostic biomarkers from high-dimensional transcriptomic data (Friedman et al., 2010; Langfelder and Horvath, 2008). Gene set enrichment analysis (GSEA) provides complementary information to traditional over-representation analysis by considering the rank order of all genes, enabling detection of coordinated pathway-level changes (Wu et al., 2021). Immune cell deconvolution methods such as MCP-counter and xCell enable characterization of the tissue immune microenvironment from bulk RNA-seq data (Aran et al., 2017; Becht et al., 2016).

In this study, we primarily focused on FCD and TSC as the most common cortical malformations, while also including TLE-HS as a comparator epileptogenic lesion to identify broader shared molecular signatures. Specific aims were: (1) identify shared DEGs across all four subtypes; (2) construct WGCNA co-expression networks and identify cross-subtype hub genes; (3) build and externally validate a multi-gene diagnostic classifier using LASSO and random forest; (4) characterize immune microenvironment alterations using multi-method deconvolution; and (5) perform GSEA, drug repurposing, and transcription factor prediction. In addition, we performed *in vitro* validation experiments to confirm the protein-level expression patterns of key hub genes under disease-relevant stimulatory conditions.

## 2 Materials and methods

### 2.1 Data acquisition and preprocessing

Transcriptomic data were obtained from the Gene Expression Omnibus (GEO)^1^. The training dataset GSE256068 comprised RNA-seq data from 85 samples in total, including 64 cortical samples (FCD2a, *n* = 18; FCD2b, *n* = 18; TSC, *n* = 14; ControlCortex, *n* = 14) and 21 hippocampal samples (TLE-HS, *n* = 8; ControlHippocampus, *n* = 13), profiled on the Illumina HiSeq platform. Clinical metadata included tissue region, sex, age, and seizure frequency (Supplementary Table 1). The external validation dataset GSE213488 included RNA-seq count data from cortical gray matter samples (FCD2a, *n* = 7; FCD2b, *n* = 6; Control, *n* = 4). Raw count matrices were normalized to log2(CPM+1) values prior to analysis. No ethical approval was required as all data were obtained from publicly available repositories. Principal component analysis of cortical samples is shown in Supplementary Figure 7.

### 2.2 Differential expression analysis

Differential expression analysis was performed using the limma package (v3.60) in R (v4.5.1) (Ritchie et al., 2015). For cortical samples, the linear model included group (ControlCortex, FCD2a, FCD2b, TSC) and tissue region (temporal, frontal, occipital, parietal) as covariates. For hippocampal samples, the model was ∼ group. Contrasts were constructed for FCD2a vs. ControlCortex, FCD2b vs. ControlCortex, TSC vs. ControlCortex, and TLE-HS vs. ControlHippocampus. DEGs were defined by |log2 fold change| > 1 and Benjamini-Hochberg adjusted *P*-value < 0.05. Gene IDs were mapped to HGNC symbols using org.Hs.eg.db (v3.19). Intersection analysis was performed using UpSetR and ggVennDiagram (Gao et al., 2021), and volcano plots were generated. As a sensitivity analysis, a three-way intersection restricted to cortical samples (FCD2a, FCD2b, and TSC), excluding hippocampal TLE-HS, was additionally performed (Supplementary Figure 6), which identified 564 shared DEGs across the three cortical groups. All 49 four-way shared DEGs remained shared among the three cortical groups, and all 15 hub genes and all 15 KEGG pathways were retained, confirming that the core immune-related signature is robust to the exclusion of TLE-HS.

### 2.3 Weighted gene co-expression network analysis

Weighted gene co-expression network analysis was performed using the WGCNA package (v1.72) (Langfelder and Horvath, 2008). The top 5,000 most variable genes (by median absolute deviation) from cortical samples were used to construct a signed co-expression network with soft-thresholding power beta = 4 (scale-free R-squared > 0.85). Modules were identified using dynamic tree cutting (deepSplit = 2, minModuleSize = 30) and merged at a cut height of 0.25. Module eigengenes were correlated with clinical traits. The complete module gene assignments are provided in Supplementary Table 3. Module membership (MM) and gene significance for TSC (GS.TSC) were calculated using TSC as a representative disease phenotype because TSC is driven by a well-defined monogenic pathway (TSC1/TSC2-mTORC1) and showed the strongest association with the brown module. Importantly, this choice does not bias the final gene set toward TSC-specific genes: hub gene candidates were additionally required to overlap with the 49 DEGs shared across all four subtypes, ensuring cross-subtype differential expression. Gene significance for FCD2a and FCD2b was also computed for the 15 hub genes (Supplementary Table 4). Hub genes were defined by: (1) |GS.TSC| > 0.5; (2) |MM| > 0.8 in brown or red modules; (3) membership in the 49 shared DEGs. *In silico* node deletion analysis (also termed virtual knockout) was performed by computing mean connectivity of the brown module TOM before and after removing each gene.

### 2.4 Functional enrichment analysis

Over-representation analysis (ORA) of Gene Ontology (GO) and KEGG pathways was performed using clusterProfiler (v4.16) with Benjamini-Hochberg correction (adjusted *P* < 0.05) (Wu et al., 2021). Gene set enrichment analysis (GSEA) was performed using clusterProfiler::gseKEGG and gseGO with gene lists ranked by log2 fold change for each comparison. KEGG pathways significantly enriched (adjusted *P* < 0.05) in all four comparisons were identified as cross-subtype common pathways (Supplementary Tables 5, 6).

### 2.5 Protein-protein interaction network

A protein-protein interaction (PPI) network was constructed using the STRING database (v12.0) with a minimum interaction score of 0.4 (Szklarczyk et al., 2023).

### 2.6 Immune cell infiltration analysis

Immune cell infiltration was assessed using MCP-counter (Becht et al., 2016) and independently cross-validated using xCell (Aran et al., 2017). Differences across groups were assessed by Kruskal-Wallis tests, and associations with clinical variables were evaluated by Spearman correlation and partial correlation analysis (ppcor package).

### 2.7 Diagnostic model construction and validation

The 15 identified hub genes were used as the input feature set for machine learning-based feature selection. LASSO logistic regression (glmnet v4.1, lambda.min, 5-fold CV) (Friedman et al., 2010) and random forest (randomForest v4.7, 500 trees) (Breiman, 2001) were applied to identify the most discriminative features. For random forest, variable importance was assessed by mean decrease in Gini impurity with ntree = 500 and default mtry (square root of the number of features). Genes selected by both algorithms were used to construct a multi-gene logistic regression model. Performance was assessed by ROC-AUC (pROC v1.19) (Robin et al., 2011). To avoid information leakage, feature selection was performed within each cross-validation fold using a nested approach: within each training fold, LASSO and random forest were applied to select features, and the resulting model was evaluated on the held-out fold. Across the five folds, CX3CR1 was selected in all five folds and P2RY12 in four of five folds, supporting their robustness as diagnostic features. The model was subsequently evaluated by internal 5-fold cross-validation and external validation on GSE213488. For cross-platform validation, expression values in both datasets were log2(CPM+1)-transformed to ensure comparability. No explicit batch correction was applied, and this is acknowledged as a limitation.

### 2.8 Drug repurposing and transcription factor prediction

Drug repurposing and transcription factor enrichment were performed using Enrichr (Chen et al., 2013) with the 15 hub genes as input, querying the DSigDB, DrugMatrix, ChEA_2022, and TRRUST databases. miRNA target prediction was performed using TargetScan and miRTarBase. Terms with adjusted *P* < 0.05 were considered significant.

### 2.9 Statistical analysis

All analyses were performed in R v4.5.1 with Bioconductor 3.20. Multiple testing was corrected using the Benjamini-Hochberg method. Group comparisons used Kruskal-Wallis or Wilcoxon rank-sum tests. Correlations were assessed using Spearman’s rho. Partial correlation controlled for age, sex, and disease subtype. *P* < 0.05 was considered significant.

For *in vitro* experiments, comparisons among the three groups (Control, LPS, MHY1485) were performed using one-way ANOVA followed by Tukey *post-hoc* test. Data are presented as mean ± SEM from three independent experiments. *P* < 0.05 was considered statistically significant.

### 2.10 Cell culture and *in vitro* validation

BV-2 murine microglial cells were cultured in DMEM supplemented with 10% fetal bovine serum at 37 °C with 5% CO_2_. Cells were divided into three groups (*n* = 3 per group): (1) Control group, treated with phosphate-buffered saline (PBS); (2) LPS group, stimulated with lipopolysaccharide (100 ng/mL, 24 h) to induce classical neuroinflammation; and (3) MHY1485 group, treated with MHY1485 (10 μM, 24 h), a potent mTORC1 activator (Laplante and Sabatini, 2012), to mimic the mTOR pathway hyperactivation characteristic of FCD and TSC (Weichhart et al., 2008).

**TABLE 1:** Top 20 differentially expressed genes per comparison.

ENSEMBL	Symbol	logFC	AveExpr	*P*-value	adj.P val	Comparison
ENSG00000232810	TNF	4.279914	−0.72045	6.44E-13	9.19E-09	FCD2a_vs_Control
ENSG00000273409	LINC02712	2.536294	−0.31745	6.48E-13	9.19E-09	FCD2a_vs_Control
ENSG00000110876	SELPLG	2.444157	4.284553	1.58E-12	1.49E-08	FCD2a_vs_Control
ENSG00000166428	PLD4	2.498512	3.660507	3.13E-12	2.22E-08	FCD2a_vs_Control
ENSG00000265206	MIR142HG	3.284463	−0.86074	5.69E-12	2.85E-08	FCD2a_vs_Control
ENSG00000132744	ACY3	3.659957	1.963171	7.00E-12	2.85E-08	FCD2a_vs_Control
ENSG00000073737	DHRS9	3.232271	2.074282	7.04E-12	2.85E-08	FCD2a_vs_Control
ENSG00000168329	CX3CR1	3.265424	5.375953	1.89E-11	6.72E-08	FCD2a_vs_Control
ENSG00000181631	P2RY13	2.957883	3.164125	2.81E-11	8.87E-08	FCD2a_vs_Control
ENSG00000161911	TREML1	2.649109	0.065319	4.98E-11	1.41E-07	FCD2a_vs_Control
ENSG00000169313	P2RY12	2.50575	5.397987	1.82E-10	4.43E-07	FCD2a_vs_Control
ENSG00000261795	NA	2.877052	−0.04476	1.87E-10	4.43E-07	FCD2a_vs_Control
ENSG00000183160	TMEM119	2.224181	3.616354	2.18E-10	4.76E-07	FCD2a_vs_Control
ENSG00000159958	TNFRSF13C	1.830269	1.66637	2.81E-10	5.69E-07	FCD2a_vs_Control
ENSG00000227039	ITGB2-AS1	2.520411	0.913236	6.83E-10	1.27E-06	FCD2a_vs_Control
ENSG00000130300	PLVAP	1.796115	1.706669	7.14E-10	1.27E-06	FCD2a_vs_Control
ENSG00000168334	XIRP1	4.292975	−0.54609	7.95E-10	1.33E-06	FCD2a_vs_Control
ENSG00000198019	NA	2.389286	1.964997	9.28E-10	1.46E-06	FCD2a_vs_Control
ENSG00000182557	SPNS3	2.423581	−0.29791	1.24E-09	1.86E-06	FCD2a_vs_Control
ENSG00000184574	LPAR5	1.935075	2.580729	1.52E-09	2.16E-06	FCD2a_vs_Control
ENSG00000110876	SELPLG	2.808067	4.284553	2.18E-17	4.23E-13	FCD2b_vs_Control
ENSG00000265206	MIR142HG	3.870079	−0.86074	3.39E-17	4.23E-13	FCD2b_vs_Control
ENSG00000232810	TNF	4.713434	−0.72045	5.37E-17	4.23E-13	FCD2b_vs_Control
ENSG00000166428	PLD4	2.862417	3.660507	5.97E-17	4.23E-13	FCD2b_vs_Control
ENSG00000132744	ACY3	4.265604	1.963171	7.46E-17	4.23E-13	FCD2b_vs_Control
ENSG00000181631	P2RY13	3.248837	3.164125	6.37E-15	3.01E-11	FCD2b_vs_Control
ENSG00000159958	TNFRSF13C	2.132935	1.66637	9.67E-15	3.92E-11	FCD2b_vs_Control
ENSG00000168329	CX3CR1	3.469769	5.375953	1.62E-14	5.74E-11	FCD2b_vs_Control
ENSG00000273409	LINC02712	2.443809	−0.31745	2.13E-14	6.72E-11	FCD2b_vs_Control
ENSG00000233695	NA	2.842343	0.78831	2.57E-14	7.30E-11	FCD2b_vs_Control
ENSG00000161911	TREML1	2.839195	0.065319	3.65E-14	9.40E-11	FCD2b_vs_Control
ENSG00000157303	SUSD3	2.473574	1.104524	9.32E-14	2.06E-10	FCD2b_vs_Control
ENSG00000213316	LTC4S	1.825637	0.830065	9.42E-14	2.06E-10	FCD2b_vs_Control
ENSG00000073737	DHRS9	3.158936	2.074282	1.63E-13	3.30E-10	FCD2b_vs_Control
ENSG00000183160	TMEM119	2.395707	3.616354	1.82E-13	3.45E-10	FCD2b_vs_Control
ENSG00000227502	MROCKI	2.135494	1.478894	3.08E-13	5.46E-10	FCD2b_vs_Control
ENSG00000182578	CSF1R	2.114895	5.859203	8.40E-13	1.36E-09	FCD2b_vs_Control
ENSG00000169313	P2RY12	2.577669	5.397987	8.90E-13	1.36E-09	FCD2b_vs_Control
ENSG00000164691	TAGAP	2.110511	1.943032	9.44E-13	1.36E-09	FCD2b_vs_Control
ENSG00000105122	RASAL3	2.346258	2.130335	9.55E-13	1.36E-09	FCD2b_vs_Control
ENSG00000166428	PLD4	3.29439	3.660507	2.35E-18	6.61E-14	TSC_vs_Control
ENSG00000110876	SELPLG	3.12095	4.284553	4.66E-18	6.61E-14	TSC_vs_Control
ENSG00000232810	TNF	5.182844	−0.72045	1.97E-17	1.86E-13	TSC_vs_Control
ENSG00000168329	CX3CR1	4.223891	5.375953	6.64E-17	4.71E-13	TSC_vs_Control
ENSG00000204472	AIF1	2.491874	3.524809	1.27E-16	6.66E-13	TSC_vs_Control
ENSG00000182578	CSF1R	2.784565	5.859203	1.64E-16	6.66E-13	TSC_vs_Control
ENSG00000183160	TMEM119	3.033668	3.616354	1.64E-16	6.66E-13	TSC_vs_Control
ENSG00000164691	TAGAP	2.738296	1.943032	3.70E-16	1.20E-12	TSC_vs_Control
ENSG00000077420	APBB1IP	2.692646	3.572623	3.79E-16	1.20E-12	TSC_vs_Control
ENSG00000169313	P2RY12	3.311423	5.397987	5.42E-16	1.54E-12	TSC_vs_Control
ENSG00000273409	LINC02712	2.840534	−0.31745	7.57E-16	1.95E-12	TSC_vs_Control
ENSG00000184574	LPAR5	2.700786	2.580729	9.82E-16	2.32E-12	TSC_vs_Control
ENSG00000181631	P2RY13	3.642049	3.164125	1.08E-15	2.35E-12	TSC_vs_Control
ENSG00000157303	SUSD3	2.940384	1.104524	1.37E-15	2.77E-12	TSC_vs_Control
ENSG00000140749	IGSF6	2.369743	2.612457	1.69E-15	3.19E-12	TSC_vs_Control
ENSG00000073737	DHRS9	3.781361	2.074282	1.85E-15	3.29E-12	TSC_vs_Control
ENSG00000247774	PCED1B-AS1	2.567951	1.27287	2.01E-15	3.35E-12	TSC_vs_Control
ENSG00000162511	LAPTM5	2.420309	5.855849	2.65E-15	4.17E-12	TSC_vs_Control
ENSG00000233695	NA	3.21573	0.78831	3.07E-15	4.43E-12	TSC_vs_Control
ENSG00000132744	ACY3	4.230594	1.963171	3.12E-15	4.43E-12	TSC_vs_Control
ENSG00000166428	PLD4	2.32499	4.082951	5.34E-23	9.59E-19	TLE_HS_vs_Control
ENSG00000215417	MIR17HG	3.606882	−1.21393	9.65E-23	9.59E-19	TLE_HS_vs_Control
ENSG00000132744	ACY3	4.026643	2.343894	1.01E-22	9.59E-19	TLE_HS_vs_Control
ENSG00000273409	LINC02712	2.292045	0.056196	2.95E-22	2.09E-18	TLE_HS_vs_Control
ENSG00000110876	SELPLG	2.16719	4.953597	6.45E-22	3.52E-18	TLE_HS_vs_Control
ENSG00000277632	CCL3	6.319497	4.486687	7.44E-22	3.52E-18	TLE_HS_vs_Control
ENSG00000267559	NA	−1.66195	0.261124	1.10E-21	4.44E-18	TLE_HS_vs_Control
ENSG00000181631	P2RY13	2.661625	3.820326	4.09E-21	1.45E-17	TLE_HS_vs_Control
ENSG00000114120	SLC25A36	0.832405	6.439806	8.55E-21	2.69E-17	TLE_HS_vs_Control
ENSG00000101335	MYL9	−2.02901	3.807287	1.11E-20	3.16E-17	TLE_HS_vs_Control
ENSG00000119608	PROX2	3.229791	−1.25299	1.51E-20	3.89E-17	TLE_HS_vs_Control
ENSG00000280234	NA	−2.21018	3.529101	2.00E-20	4.72E-17	TLE_HS_vs_Control
ENSG00000232810	TNF	6.546365	1.638046	2.29E-20	4.99E-17	TLE_HS_vs_Control
ENSG00000083642	PDS5B	−1.44579	6.486845	2.51E-20	5.10E-17	TLE_HS_vs_Control
ENSG00000235613	NSRP1P1	−3.67445	−0.43995	3.49E-20	6.59E-17	TLE_HS_vs_Control
ENSG00000287258	LOC124901391	−2.51022	2.06901	1.04E-19	1.78E-16	TLE_HS_vs_Control
ENSG00000196659	IFT70B	1.071086	2.176478	1.13E-19	1.78E-16	TLE_HS_vs_Control
ENSG00000260691	ANKRD20A1	−2.96029	2.690098	1.14E-19	1.78E-16	TLE_HS_vs_Control
ENSG00000230606	NA	−1.9791	4.724342	1.19E-19	1.78E-16	TLE_HS_vs_Control
ENSG00000198467	TPM2	−1.88788	4.662063	2.06E-19	2.92E-16	TLE_HS_vs_Control

Total RNA was extracted using TRIzol reagent (Invitrogen) and reverse-transcribed into cDNA using the PrimeScript RT reagent kit (TaKaRa). Quantitative real-time PCR (qPCR) was performed using SYBR Green Master Mix (TaKaRa) on an Applied Biosystems QuantStudio 6 Flex system. Primer sequences, sourced from PrimerBank and published literature, were as follows: Irf8 (Forward: 5′-CAGGTGGGCAAGATCCAGAA-3′, Reverse: 5′-TGGTGAGGAAGTTGGCGATA-3′), Cx3cr1 (Forward: 5′-CCATCTGCTCAGGACCTCAC-3′, Reverse: 5′-CA CCAGACACTCGCCATTTCT-3′), P2ry12 (Forward: 5′-TGCCAT CTGTACCTGCCTTC-3′, Reverse: 5′-CGTCCCTTTTCCCCAT CCAA-3′), Tlr7 (Forward: 5′-GGAAATTGCCCTCGATGTTA-3′, Reverse: 5′-CAAAAATTTGGCCTCCTCAA-3′), and Gapdh (Forward: 5′-AGGTCGGTGTGAACGGATTTG-3′, Reverse: 5′-TGTAGACCATGTAGTTGAGGTCA-3′). Relative mRNA expression was calculated using the 2 to the power of negative delta Ct method with Gapdh as the internal reference.

For Western blot analysis, total protein was extracted using RIPA lysis buffer (Beyotime) containing protease and phosphatase inhibitors, separated by 10% SDS-PAGE, and transferred to PVDF membranes (Millipore). Membranes were blocked with 5% non-fat milk and incubated overnight at 4 °C with the following primary antibodies: anti-IRF8 (1:1000, Proteintech, 18977-1-AP), anti-CX3CR1 (1:500, Proteintech, 29819-1-AP), anti-P2RY12 (1:500, Proteintech, 11976-1-AP), anti-phospho-S6 (Ser240/244) (1:1000, Proteintech, 29220-1-AP), and anti-GAPDH (1:5000, Proteintech, 60004-1-Ig). After washing, membranes were incubated with HRP-conjugated secondary antibodies (1:5000, Proteintech) for 1 h at room temperature. Protein bands were visualized by enhanced chemiluminescence (ECL, Beyotime) and quantified using ImageJ. All experiments were performed using three independent biological replicates (*n* = 3), each with technical duplicates.

## 3 Results

### 3.1 Forty-nine shared DEGs across four epilepsy subtypes

Differential expression analysis identified substantial transcriptomic alterations: 865 DEGs (FCD2a vs. Control), 1,915 DEGs (FCD2b vs. Control), 2,772 DEGs (TSC vs. Control), and 3,749 DEGs (TLE-HS vs. Control) (Figures 1A–D, Supplementary Figure 1, and Table 1). The majority of DEGs were upregulated. Four-way intersection analysis revealed 49 shared DEGs across all subtypes (Figures 1E, F and Supplementary Table 2), all consistently upregulated, indicating a conserved activation signature.

**FIGURE 1:**
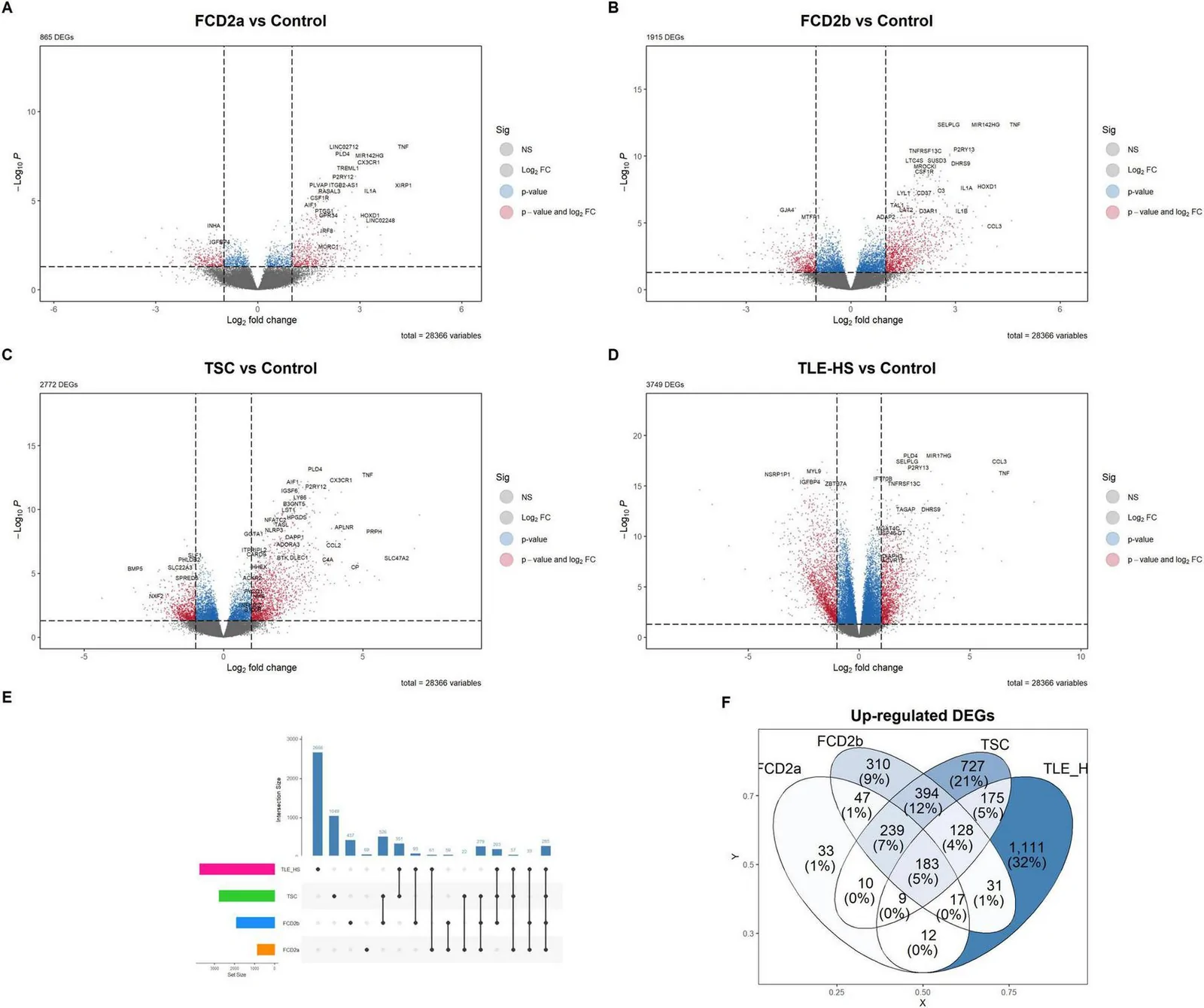
Differential expression analysis across epilepsy subtypes. **(A–D)** Volcano plots for FCD2a, FCD2b, TSC vs. Control, and TLE-HS vs. Control. Red: upregulated; blue: downregulated (|log2FC| > 1, adjusted *P* < 0.05). **(E)** UpSet plot of four-way DEG intersection. **(F)** Venn diagram of shared DEGs.

### 3.2 WGCNA reveals disease-associated immune modules

Weighted gene co-expression network analysis of cortical samples (beta = 4, signed network) identified the brown module (MEbrown) and red module (MEred) as the modules most significantly associated with disease status (Figure 2 and Supplementary Figure 3). Gene Ontology enrichment of brown module genes revealed predominant involvement in immune system processes, inflammatory response, and myeloid leukocyte activation.

**FIGURE 2:**
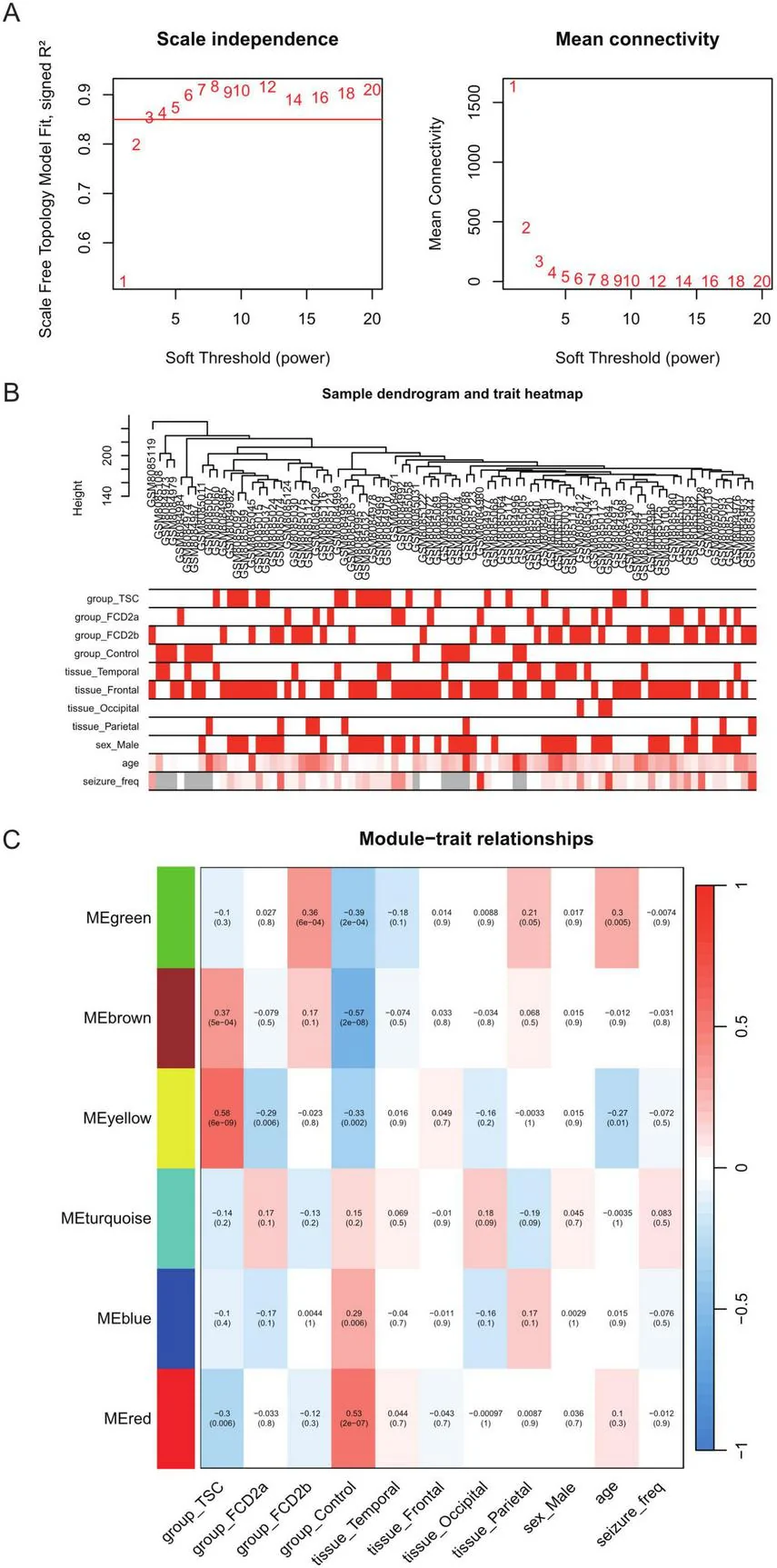
Weighted gene co-expression network analysis (WGCNA) module identification. **(A)** Scale-free topology fit and mean connectivity across soft-thresholding powers (selected beta = 4). **(B)** Sample dendrogram with clinical trait heatmap. **(C)** Module-trait correlation heatmap.

### 3.3 Fifteen hub genes converge on microglial identity

Hub gene screening by GS, MM, and DEG overlap identified 15 hub genes, all functionally associated with microglia/myeloid lineage: IRF8, CLEC7A, CYBB, TLR7, CX3CR1, CCR1, C3AR1, P2RY12, IRF5, CD300C, GPR34, MPEG1, RGS18, LILRA2, and CD84 (Figure 3A–C; Table 2). CX3CR1 and P2RY12 are canonical homeostatic microglial markers (Hickman et al., 2013). IRF8 is a master transcription factor for microglial development (Kierdorf et al., 2013). TLR7 functions as an endosomal innate immune sensor. C3AR1 mediates complement C3a signaling (Stevens et al., 2007). IRF5 and CYBB drive inflammatory effector functions (Takaoka et al., 2005). This highly non-random functional convergence implicates a conserved microglia/myeloid-associated program in epileptic cortex.

**FIGURE 3:**
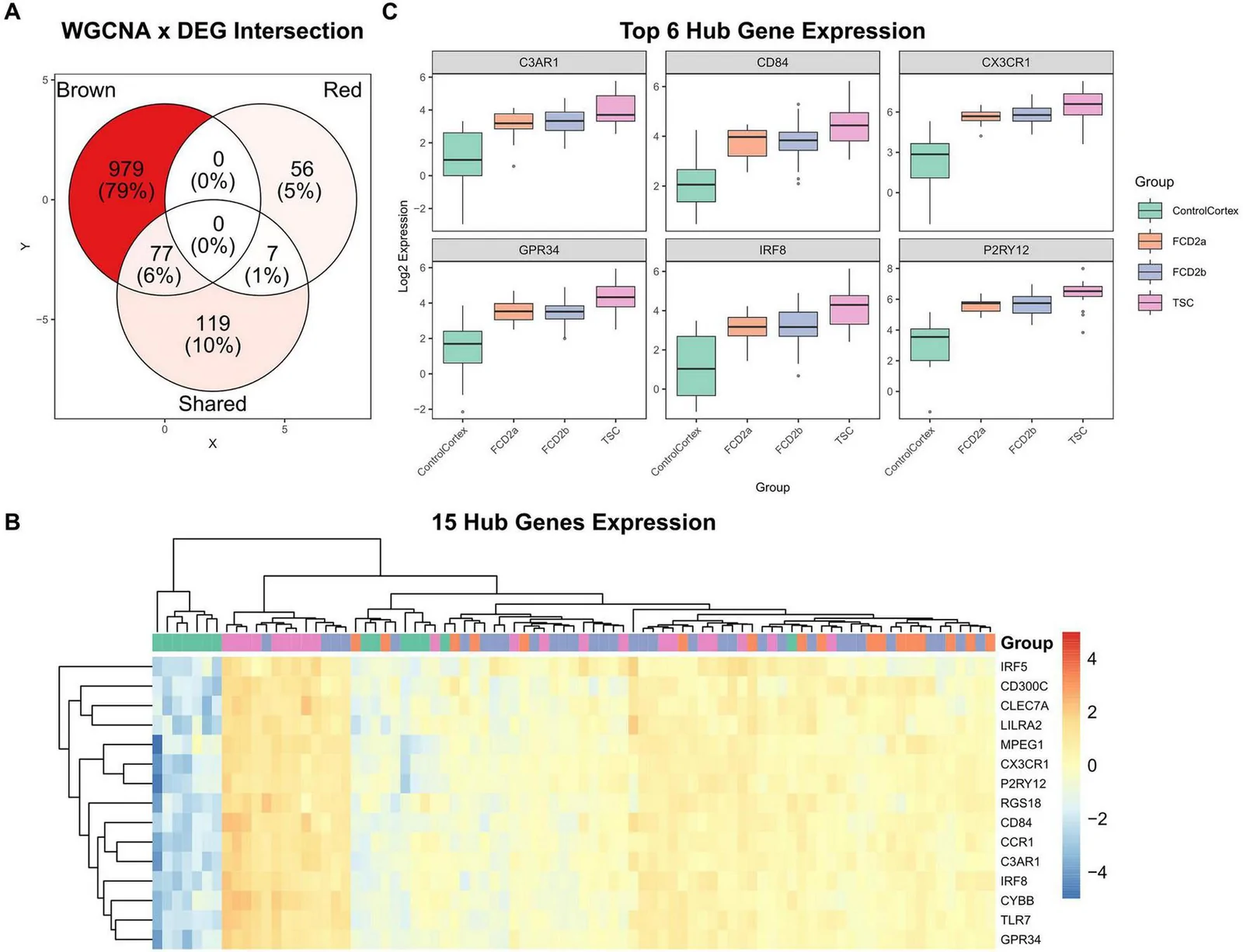
Hub gene identification and characterization. **(A)** Three-way intersection (WGCNA hub × shared DEGs × |GS|/|MM| filtering). **(B)** Expression heatmap of 15 hub genes. **(C)** Boxplots of top hub genes across subtypes.

**TABLE 2:** Fifteen hub genes identified by WGCNA and three-way DEG overlap.

Symbol	ENSEMBL	GS_TSC	GS_pvalue	MM	MM_pvalue	Module
IRF5	ENSG00000128604	0		0.427		Brown
CYBB	ENSG00000165168	0		0.228		Brown
CCR1	ENSG00000163823	0		0.333		Brown
CD84	ENSG00000066294	0		0.367		Brown
IRF8	ENSG00000140968	0		0.337		Brown
TLR7	ENSG00000196664	0		0.344		Brown
GPR34	ENSG00000171659	0		0.313		Brown
C3AR1	ENSG00000171860	0		0.452		Brown
P2RY12	ENSG00000169313	0		0.219		Brown
RGS18	ENSG00000150681	0		0.324		Brown
CX3CR1	ENSG00000168329	0		0.347		Brown
MPEG1	ENSG00000197629	0		0.247		Brown
LILRA2	ENSG00000239998	0		0.492		Brown
CD300C	ENSG00000167850	0		0.329		Brown
CLEC7A	ENSG00000172243	0		0.475		Brown

### 3.4 GSEA reveals convergent innate immune pathway enrichment

GSEA identified 15 KEGG pathways significantly enriched across all four comparisons (Figure 4, Table 3, and Supplementary Figures 2, 4, 5). The shared pathways clustered into six biological themes: (1) innate immune sensing (Toll-like receptor, NOD-like receptor, C-type lectin receptor signaling); (2) inflammatory signal transduction (NF-kB, JAK-STAT, TNF signaling); (3) immune cell communication (cytokine-cytokine receptor interaction, chemokine signaling); (4) phagocytosis (phagosome, Fc-gamma-R-mediated phagocytosis); (5) complement and coagulation cascades; and (6) hematopoietic/immune cell biology (hematopoietic cell lineage, osteoclast differentiation, Th17 differentiation). Notably, three hub genes directly participate in these pathways: TLR7 in TLR signaling, IRF5/IRF8 in NF-kB signaling, and CCR1/CX3CR1 in chemokine signaling.

**FIGURE 4:**
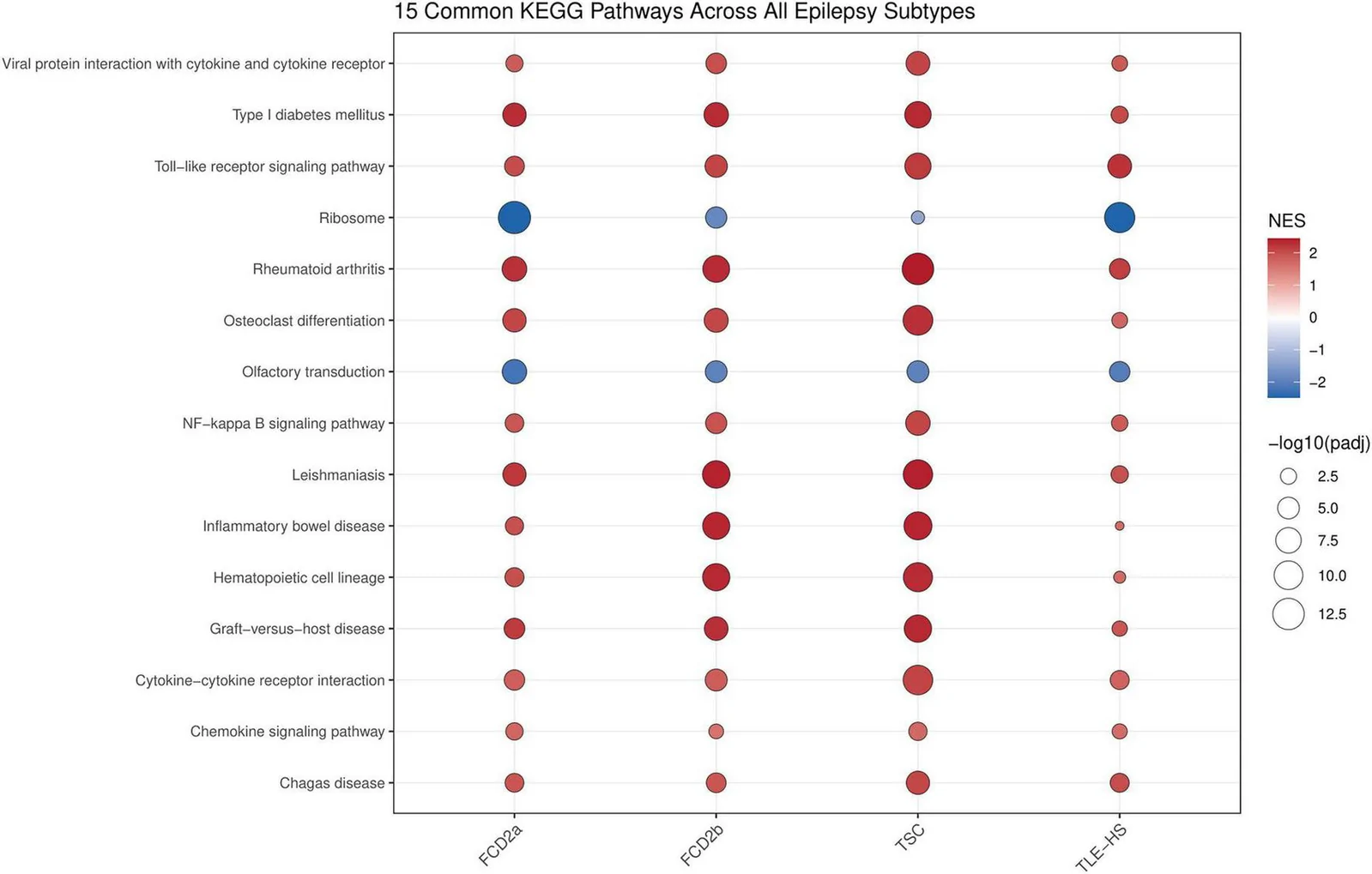
Bubble plot of 15 KEGG pathways significantly enriched across all four comparisons. Size: −log10(adjusted P); color: NES.

**TABLE 3:** Fifteen common KEGG pathways significantly enriched across all four comparisons (NES and adjusted *P*-value).

Pathway ID	Pathway description	FCD2a NES	FCD2b NES	TSC NES	TLE-HS NES
hsa03010	Ribosome	−2.46***	−1.85***	−1.37*	−2.46***
hsa05323	Rheumatoid arthritis	2.27***	2.32***	2.44***	2.15***
hsa04740	Olfactory transduction	−2.18***	−1.96***	−1.95***	−2.02***
hsa04940	Type I diabetes mellitus	2.31***	2.33***	2.35***	2.04**
hsa04380	Osteoclast differentiation	2.07***	2.06***	2.29***	1.75**
hsa05140	Leishmaniasis	2.21***	2.39***	2.40***	1.97**
hsa05332	Graft-versus-host disease	2.19***	2.28***	2.35***	1.91**
hsa04060	Cytokine-cytokine receptor interaction	1.82***	1.84***	2.09***	1.78***
hsa04620	Toll-like receptor signaling pathway	2.01***	2.09***	2.19***	2.27***
hsa04640	Hematopoietic cell lineage	1.98***	2.35***	2.33***	1.68*
hsa05142	Chagas disease	1.92***	1.90***	2.06***	2.00***
hsa04064	NF-kappa B signaling pathway	1.89***	1.94***	2.07***	1.86**
hsa05321	Inflammatory bowel disease	1.97***	2.36***	2.37***	1.70*
hsa04062	Chemokine signaling pathway	1.75**	1.59**	1.70***	1.67**
hsa04061	Viral protein interaction with cytokine and cytokine receptor	1.85**	1.99***	2.10***	1.87**

NES, normalized enrichment score from gene set enrichment analysis. *P < 0.05, **P < 0.01, ***P < 0.001 (adjusted P-value, Benjamini-Hochberg method). Negative NES values indicate downregulation in the disease group.

**TABLE 4:** Per-gene AUC values across training comparisons and external validation.

Gene	FCD2a_vs_Control	FCD2b_vs_Control	TSC_vs_Control	GSE213488
IRF8	0.87395	0.852814	0.945578	0.519
CLEC7A	0.865546	0.906926	0.928571	0.731
CYBB	0.815126	0.811688	0.908163	0.587
TLR7	0.87395	0.876623	0.945578	0.567
CX3CR1	0.978992	0.97619	0.979592	0.673
CCR1	0.836134	0.839827	0.942177	0.538
C3AR1	0.852941	0.883117	0.952381	0.433
P2RY12	0.970588	0.958874	0.979592	0.846
IRF5	0.827731	0.935065	0.97619	0.471
CD300C	0.915966	0.887446	0.928571	0.731
GPR34	0.920168	0.904762	0.962585	0.731
MPEG1	0.852941	0.911255	0.931973	0.808
RGS18	0.726891	0.757576	0.87415	0.596
LILRA2	0.777311	0.87013	0.931973	0.596
CD84	0.915966	0.911255	0.97619	0.798

### 3.5 Myeloid and T-cell infiltration correlates with clinical features

MCP-counter analysis revealed significantly elevated Monocytic lineage and T cell scores in FCD2a, FCD2b, and TSC compared to ControlCortex (Figure 5A). xCell independently confirmed monocyte/macrophage and dendritic cell expansion (Figure 5D). Full xCell immune scores are provided in Supplementary Table 7. Exploratory analyses showed that monocytic lineage scores showed a non-significant positive trend with seizure frequency in FCD2a (Spearman rho = 0.478, *P* = 0.052; Figure 5B). No correction for multiple comparisons was applied to these exploratory immune-cell/clinical-variable correlation analyses, and T cell scores showed a significant negative correlation with patient age (Spearman rho = −0.373, *P* = 1.34e-3; Figure 5C).

**FIGURE 5:**
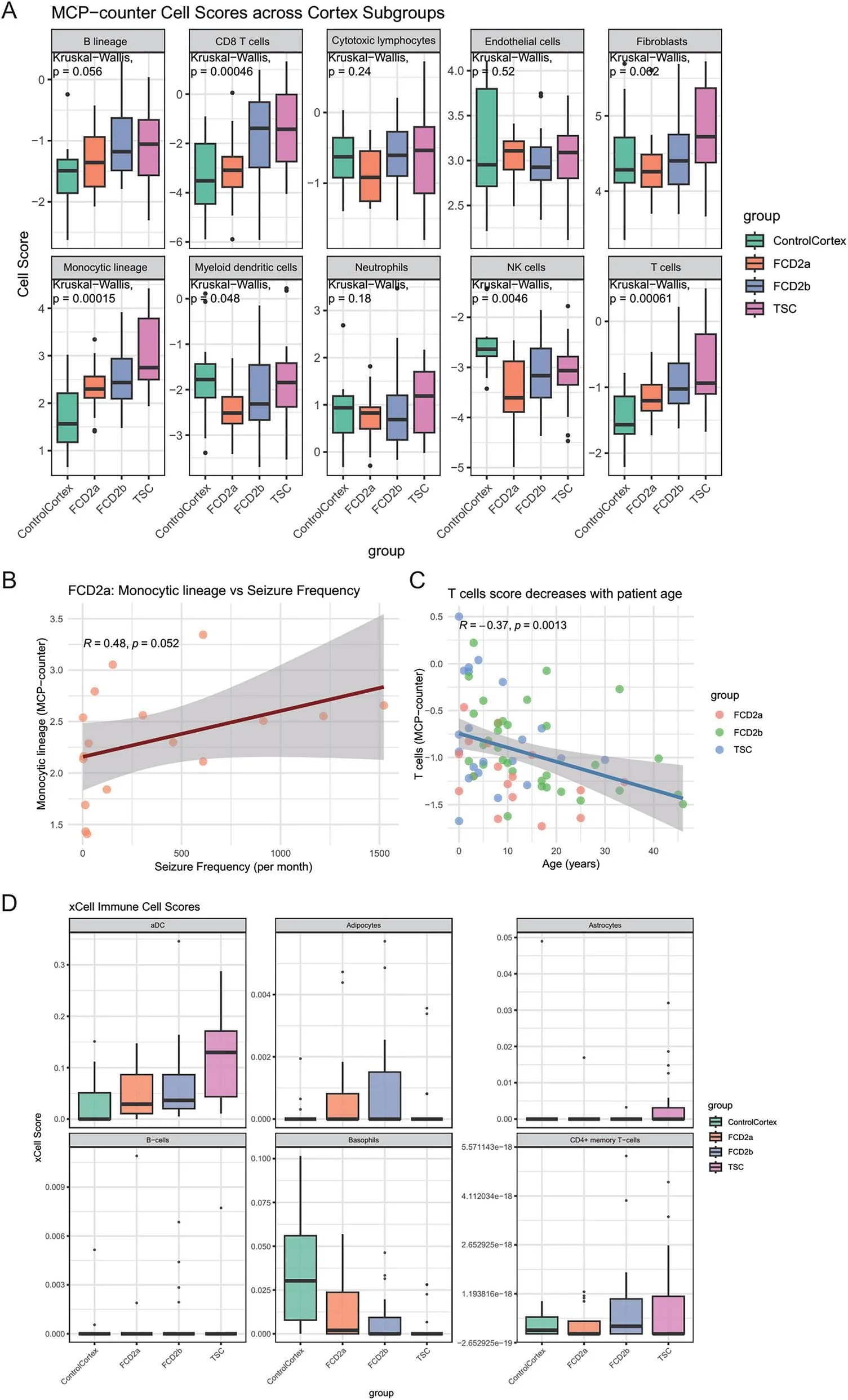
Immune cell infiltration. **(A)** MCP-counter cell scores. **(B)** Monocytic lineage vs. seizure frequency in FCD2a. **(C)** T cells vs. patient age. **(D)** xCell immune scores.

### 3.6 PPI network and virtual knockout analysis

STRING analysis revealed a dense PPI network centered on an IRF8-TLR7-CX3CR1 axis (Figure 6A). While the PPI network highlights the central connectivity of the IRF8-TLR7-CX3CR1 axis, *in silico* node deletion analysis additionally identified CLEC7A, LILRA2, and IRF5 (followed by C3AR1 and CD84) as the hub genes whose removal caused the largest decreases in brown module connectivity (Figure 6B), providing independent evidence of their structural importance.

**FIGURE 6:**
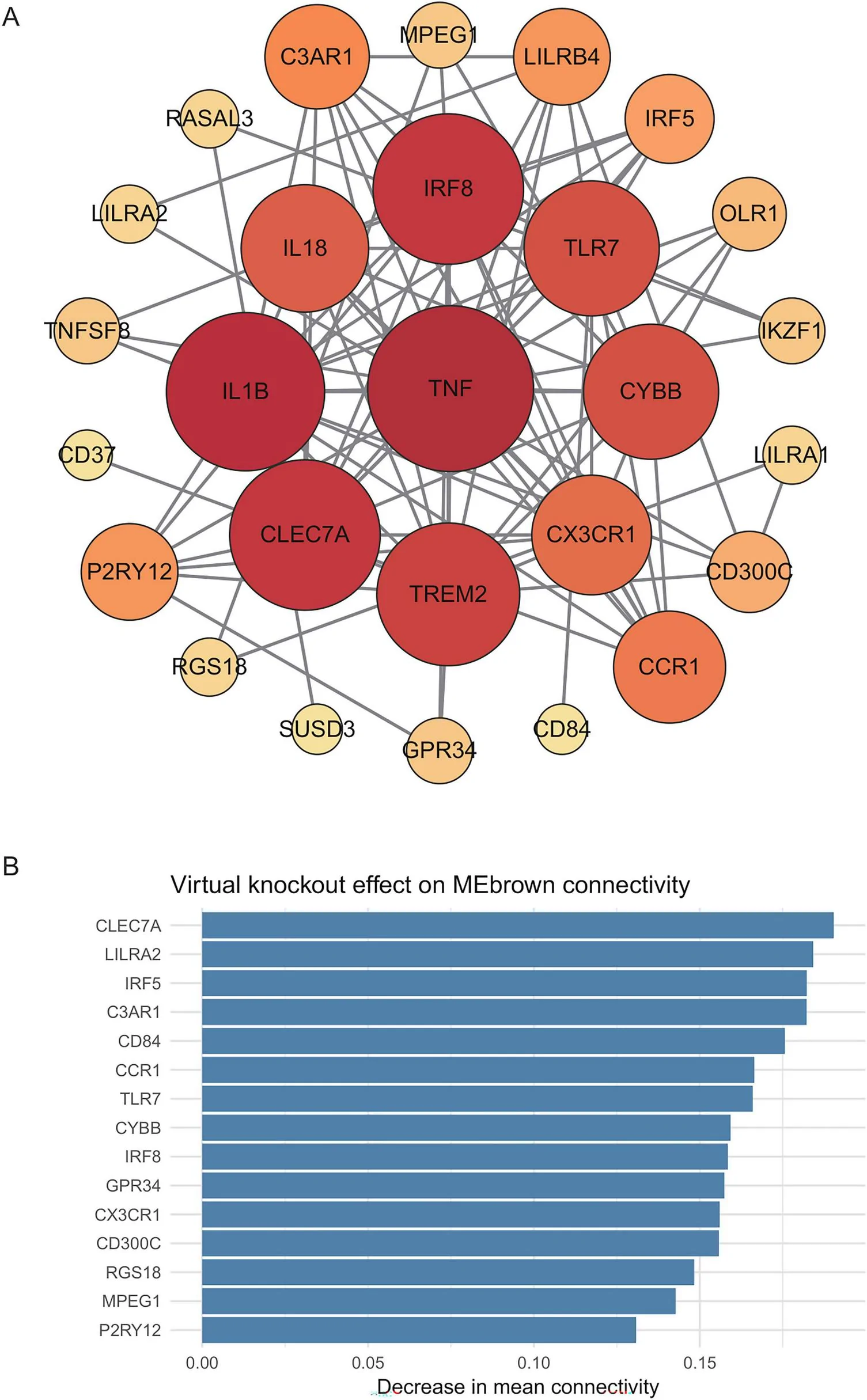
Protein-protein interaction (PPI) network and virtual knockout. **(A)** STRING PPI network of 15 hub genes. **(B)** *In silico* node deletion effect on MEbrown connectivity.

### 3.7 CX3CR1 + P2RY12 two-gene diagnostic model

Individual per-gene ROC curves for all 15 hub genes are shown in Figure 7A. LASSO selected five genes (CX3CR1, C3AR1, P2RY12, IRF5, RGS18), and random forest ranked CX3CR1, P2RY12, GPR34, CD84, and CD300C as the top five features (Figures 7B, C). Algorithm intersection yielded CX3CR1 and P2RY12. A logistic regression model incorporating these two genes achieved an AUC of 0.979 (95% CI: 0.947–1.000) in the training set (Figure 7D). Nested five-fold cross-validation, with feature selection performed within each training fold, yielded an AUC of 0.862 (95% CI: 0.693–1.000), an accuracy of 94.12%, a sensitivity of 97.18%, and a specificity of 78.57%. Across folds, CX3CR1 was selected in all five folds and P2RY12 in four of five folds. External validation on GSE213488 demonstrated an AUC of 0.865 (95% CI: 0.681–1.000, DeLong method; Figure 7E). Per-gene AUC values are reported in Table 3.

**FIGURE 7:**
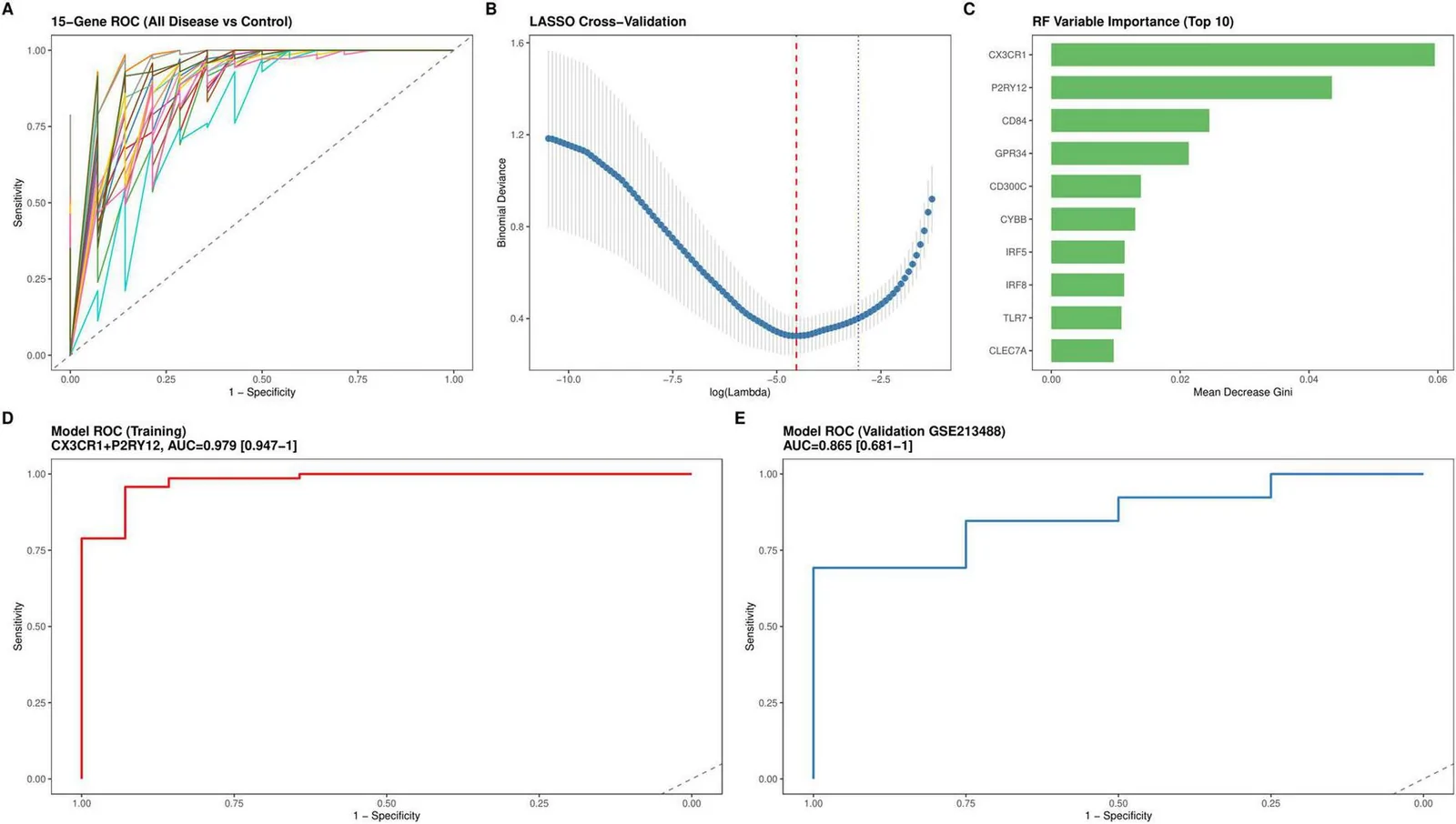
Diagnostic model construction and validation. **(A)** Multi-gene ROC curves. **(B)** LASSO cross-validation. **(C)** Random forest variable importance. **(D)** CX3CR1 + P2RY12 model ROC (training). **(E)** External validation ROC (GSE213488).

### 3.8 Drug repurposing and transcription factor prediction

Enrichr analysis identified vorinostat (SAHA), an FDA-approved HDAC inhibitor with blood-brain barrier permeability, as a hypothesis-generating drug candidate (adjusted *P* = 0.042; Figure 8A). This finding is consistent with preclinical studies demonstrating that HDAC inhibition restores synaptic plasticity and seizure threshold in TSC models (Basu et al., 2019). Additional candidates included piperlongumine, emetine, tamibarotene, and ciclopirox. IRF8 was predicted as the top upstream transcription factor (adjusted *P* = 1.45E-7; Figure 8B), consistent with its dual role as hub gene and master regulator of monocyte/microglial differentiation (Kurotaki et al., 2013).

**FIGURE 8:**
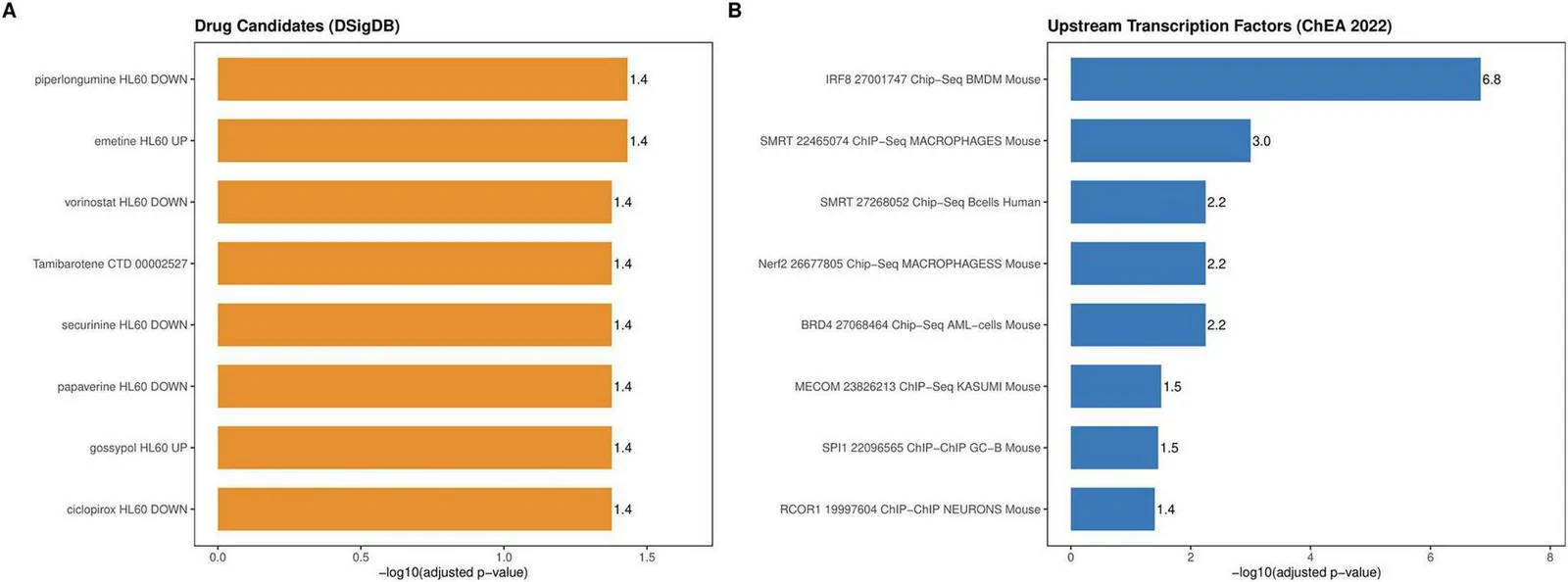
Drug repurposing and TF prediction. **(A)** Top drug candidates (DSigDB). **(B)** Top upstream transcription factors (ChEA_2022).

### 3.9 *In vitro* validation in LPS-stimulated and mTOR-activated BV-2 microglia

To experimentally validate the transcriptomic findings, we examined the expression of four key hub genes in BV-2 microglial cells under two distinct activation paradigms: classical TLR4-mediated inflammation (LPS) and mTOR-driven activation (MHY1485). qPCR analysis revealed that LPS stimulation significantly upregulated Irf8 and Tlr7 mRNA, consistent with their known roles in innate immune activation (Figure 9). Notably, P2ry12 mRNA expression was significantly downregulated in the LPS group compared to Control, whereas it was maintained or slightly upregulated in the MHY1485 group. Cx3cr1 showed a decrease under LPS stimulation but an increase under MHY1485 treatment, mirroring the divergent regulation observed for P2ry12.

**FIGURE 9:**
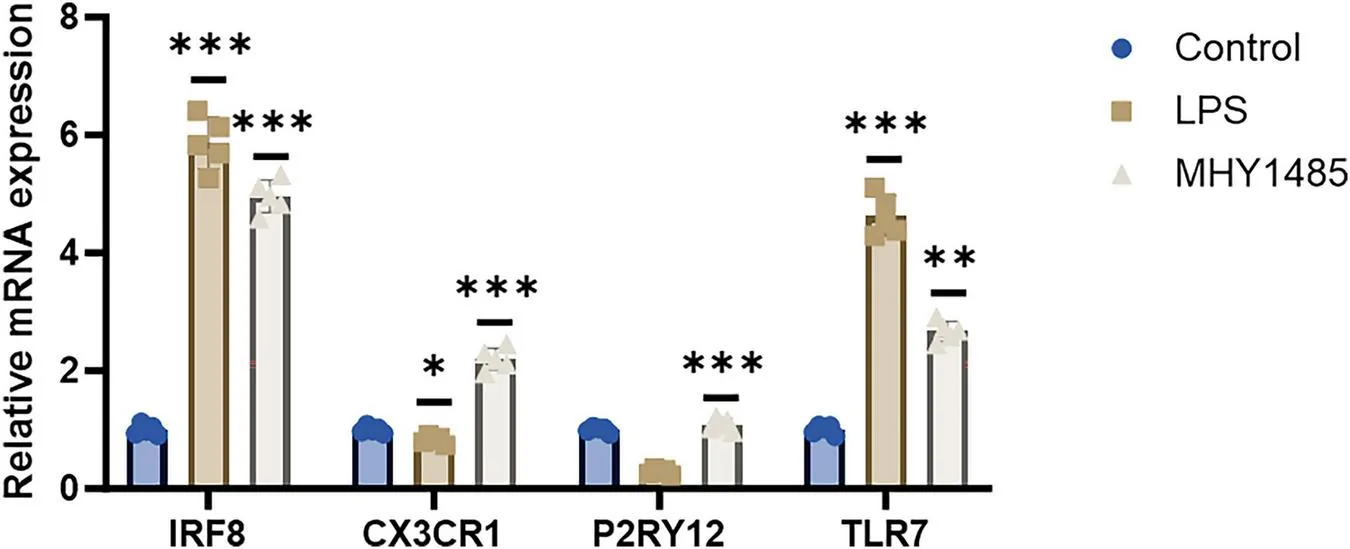
*In vitro* qPCR validation in BV-2 microglia. Relative mRNA expression of Irf8, Tlr7, P2ry12, and Cx3cr1 in Control, LPS (100 ng/mL, 24 h), and MHY1485 (10 μM, 24 h) groups. Data are presented as mean ± SEM (*n* = 3). *P < 0.05, **P < 0.01, ***P < 0.001 vs. Control; #*P* < 0.05 LPS vs. MHY1485 (one-way ANOVA with Tukey *post-hoc* test).

Western blot analysis confirmed the qPCR findings at the protein level (Figure 10). Irf8 protein was markedly upregulated in both LPS and MHY1485 groups. P2ry12 protein showed divergent regulation: decreased in the LPS group but maintained in MHY1485-treated cells. Phospho-S6 (p-S6) levels were strongly elevated in the MHY1485 group, confirming successful mTORC1 activation, but remained unchanged in the LPS group. These results provide proof-of-concept evidence that mTOR-driven activation preserves P2ry12 expression, whereas classical TLR4-mediated inflammation triggers its loss, providing mechanistic evidence that the homeostatic-to-reactive microglial transition differs fundamentally between these two activation modes.

**FIGURE 10:**
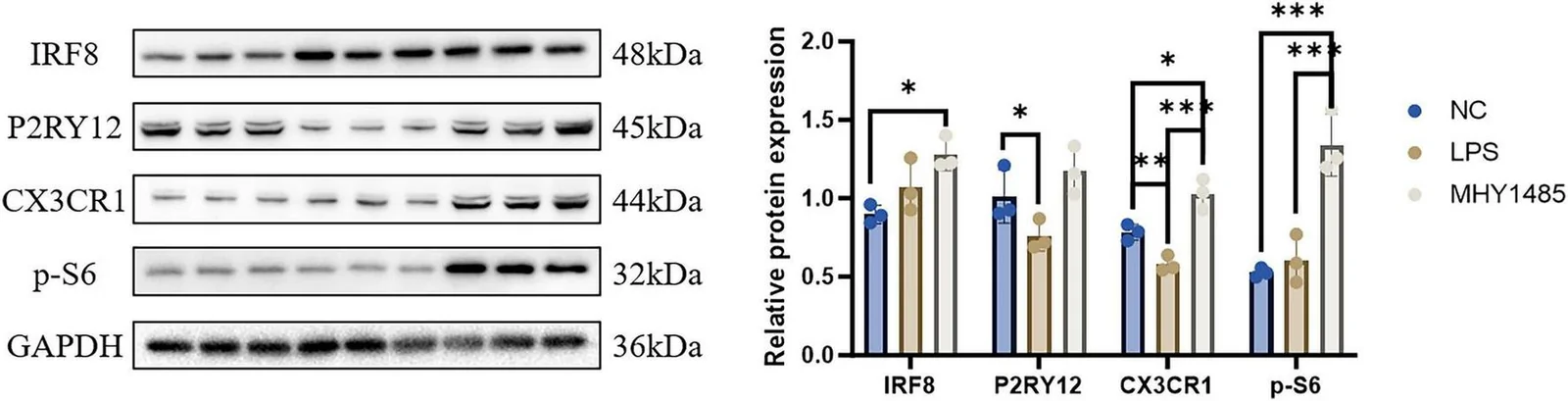
Western blot validation in BV-2 microglia. Representative Western blot images of Irf8, Cx3cr1, P2ry12, p-S6 (Ser240/244), and GAPDH across the three groups (left), and densitometric quantification of band intensities normalized to GAPDH (right). Data are presented as mean ± SEM (*n* = 3). *P < 0.05, **P < 0.01, ***P < 0.001 vs. Control.

## 4 Discussion

In this study, we identified a conserved transcriptomic program of innate immune activation, predominantly microglia/myeloid-associated, across FCD2a, FCD2b, and TSC. The 15 hub genes, all microglia/myeloid-associated, represent a cross-subtype disease module. Several genes warrant focused discussion.

CX3CR1 and P2RY12 are canonical homeostatic microglial markers (Hickman et al., 2013). Although often described as homeostatic markers, both CX3CR1 and P2RY12 can remain highly expressed in certain activated human microglial states, and their marked upregulation in patient tissue likely reflects an expanded microglial population rather than simple per-cell downregulation. This interpretation is supported by our *in vitro* experiments, which demonstrated that P2ry12 expression was preserved or upregulated under mTOR-driven activation (MHY1485) but was markedly reduced under classical TLR4-mediated inflammation (LPS; Figures 9, 10). These findings are consistent with the established role of P2RY12 in regulating microglial activation states through purinergic signaling (Haynes et al., 2006), and suggest that mTOR pathway hyperactivation, a hallmark of FCD and TSC, drives a distinct microglia/myeloid-associated program that retains features of homeostatic identity. The IRF8-IRF5-TLR7 axis represents a compelling regulatory cascade: IRF8 as master TF (Kierdorf et al., 2013), TLR7 as endogenous RNA sensor (Kawai and Akira, 2010), and IRF5 as pro-inflammatory effector (Takaoka et al., 2005). C3AR1 and complement pathway enrichment implicate complement-mediated synaptic pruning in epileptic network reorganization (Stevens et al., 2007).

Limitations include: retrospective design with public datasets; limited external validation sample size; lack of batch effect correction between training and validation datasets; and the inherent inability of bulk RNA-seq to resolve microglial abundance, activation state, or infiltrating myeloid cells. Cross-region differences between hippocampal TLE-HS and cortical FCD/TSC samples may also contribute to the observed signature, although a three-cortical-group sensitivity analysis confirmed robustness. While *in vitro* validation using the BV-2 murine microglial cell line provided protein-level confirmation of key hub genes, these findings require further validation in human iPSC-derived microglia or primary human surgical specimens. Additionally, drug predictions remain purely computational and require experimental confirmation. Future directions include scRNA-seq validation, IHC confirmation in surgical specimens, and functional studies using conditional knockout models.

## 5 Conclusion

Focal cortical dysplasia and TSC share a conserved microglia/myeloid-associated innate immune transcriptomic program. Fifteen hub genes represent a cross-subtype disease module, and CX3CR1 + P2RY12 provide a two-gene diagnostic classifier with encouraging but preliminary performance (training AUC 0.979; nested CV AUC 0.862; external validation AUC 0.865). GSEA reveals convergent TLR, NF-kB, chemokine, and complement pathway enrichment. IRF8 is the predicted master transcriptional regulator, and vorinostat is a candidate therapeutic. These findings support microglia-targeted strategies as a trans-subtype therapeutic approach in drug-resistant epilepsy.

## Data Availability

The data are publicly available in the GEO repository under accession numbers GSE213488 and GSE213488.

## Footnotes

1
https://www.ncbi.nlm.nih.gov/geo/

## Supplementary material

Supplementary Figure 1 Per-group multi-gene ROC curves.

Supplementary Figure 2 GO enrichment dotplots for 49 shared DEGs.

Supplementary Figure 3 WGCNA module eigengene dendrogram.

Supplementary Figure 4 Hub gene expression in GSE213488 validation cohort.

Supplementary Figure 5 GSEA GO BP dotplots for all comparisons.

Supplementary Figure 6 Cortical-only DEG overlap among FCD2a, FCD2b, and TSC (Venn diagram).

Supplementary Figure 7 Principal component analysis of cortical samples by disease group.

Supplementary Table 1 Sample clinical characteristics (GSE256068).

Supplementary Table 2 Full list of 49 shared DEGs.

Supplementary Table 3 WGCNA module gene assignments (MEbrown and MEred).

Supplementary Table 4 Gene significance (GS) of the 15 hub genes for TSC, FCD2a, and FCD2b.

Supplementary Table 5 Complete GSEA KEGG results for the four comparisons.

Supplementary Table 6 Complete GSEA GO BP results for the four comparisons.

Supplementary Table 7 xCell immune scores.
