# Supplementary material for: Identification of microglia-associated hub genes and a CX3CR1/P2RY12 two-gene diagnostic model for cross-subtype epilepsy-associated cortical malformations: integrated transcriptomic analysis with external validation
Source: Front Neurosci. 2026 Oct 8;20:1954412. doi: 10.3389/fnins.2026.1954412 (PMC13687088; doi:10.3389/fnins.2026.1954412)
Supplement: Supplementary file 1 [file Image_6.pdf]

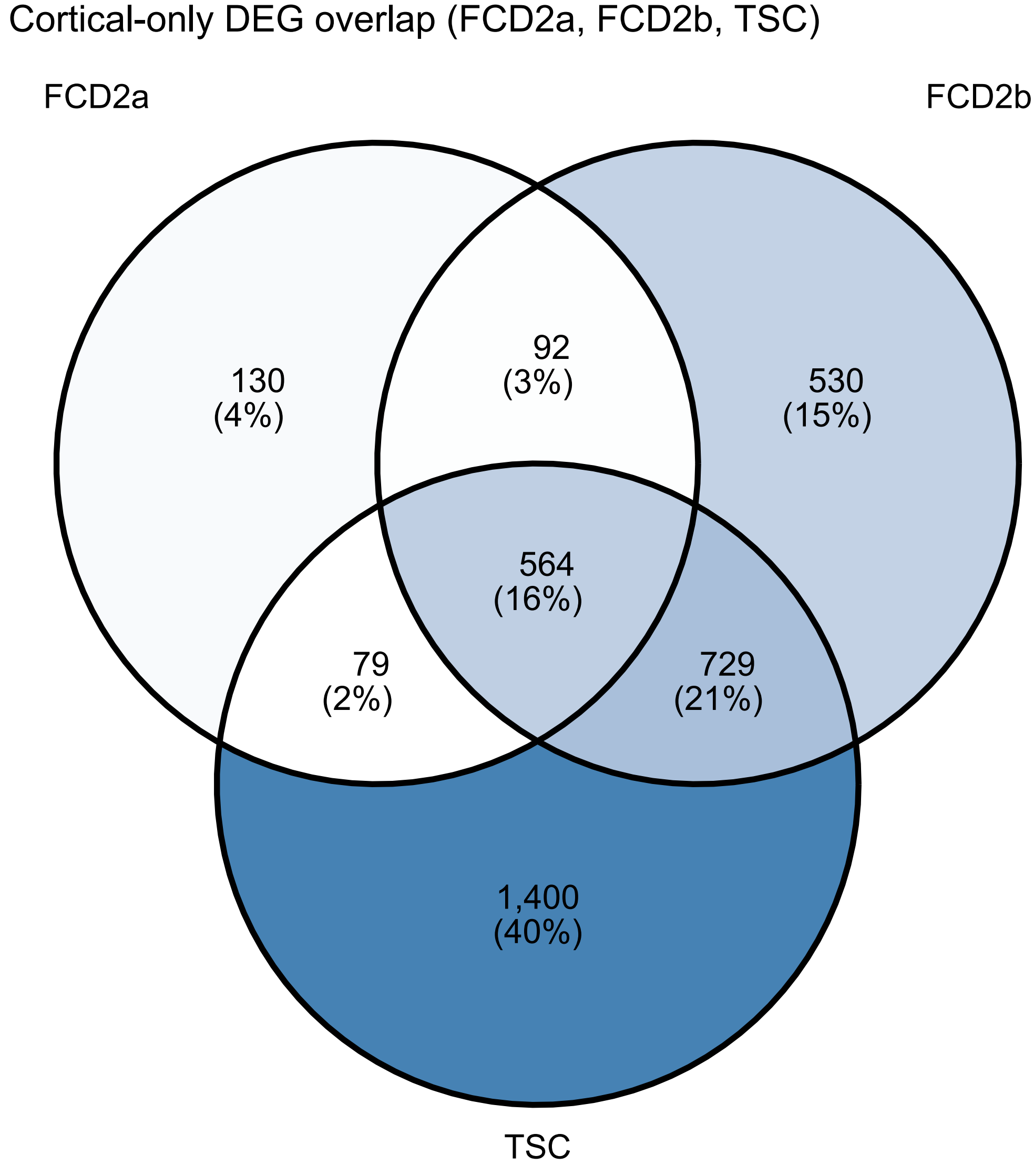
Cortical-only DEG overlap (FCD2a, FCD2b, TSC)
FCD2a
FCD2b
130
(4%)
92
(3%)
530
(15%)
564
(16%)
79
(2%)
729
(21%)
1,400
(40%)
TSC
