# Supplementary material for: Identification of microglia-associated hub genes and a CX3CR1/P2RY12 two-gene diagnostic model for cross-subtype epilepsy-associated cortical malformations: integrated transcriptomic analysis with external validation
Source: Front Neurosci. 2026 Oct 8;20:1954412. doi: 10.3389/fnins.2026.1954412 (PMC13687088; doi:10.3389/fnins.2026.1954412)
Supplement: Supplementary file 2 [file Image_7.pdf]

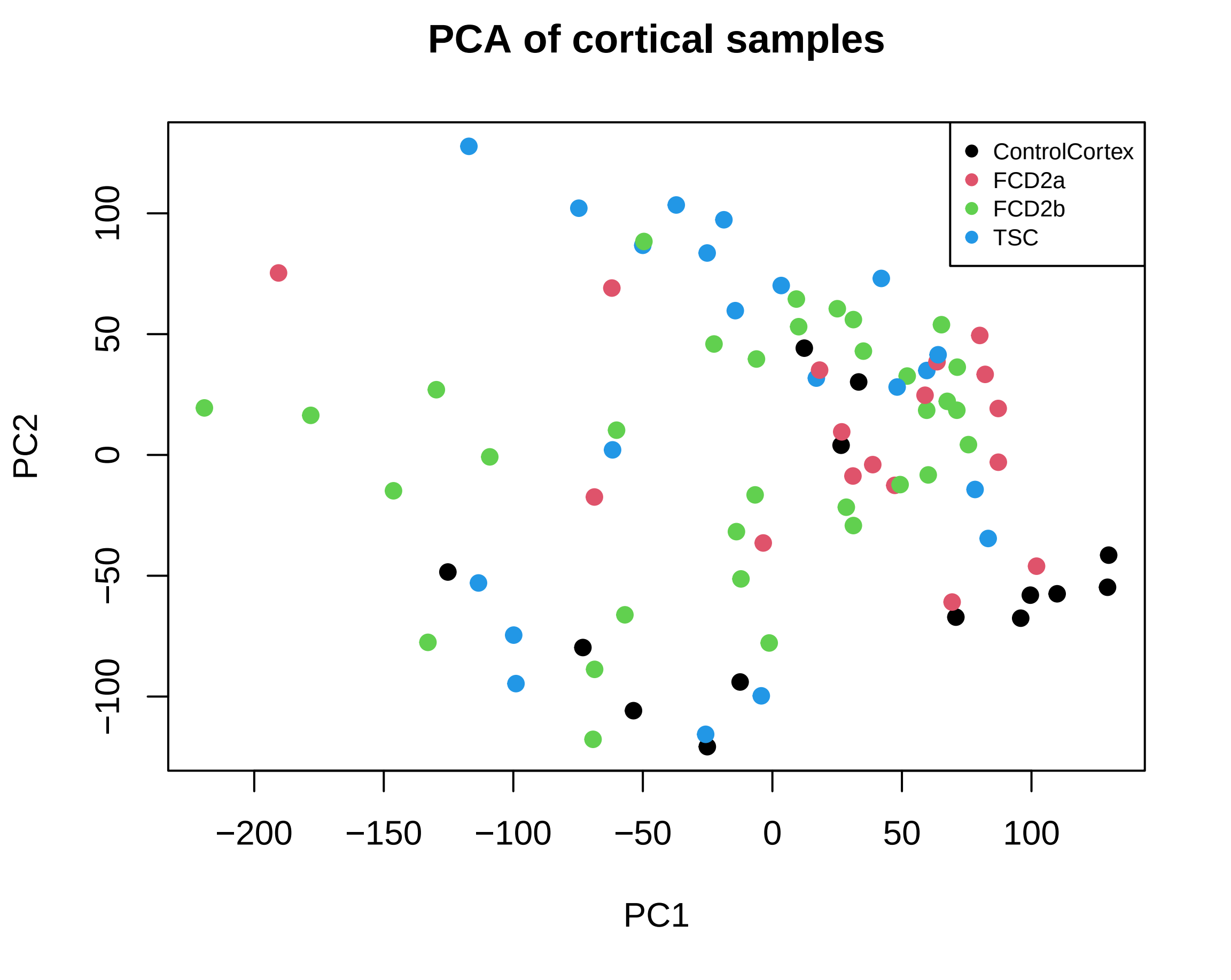
PCA of cortical samples
ControlCortex
FCD2a
FCD2b
TSC
PC1
PC2
−200
−150
−100
−50
0
50
100
